# Synthesis, Characterization, and Cytotoxic Activity of New Lanthanum(III) Complexes of Bis-Coumarins

**DOI:** 10.1155/BCA/2006/25651

**Published:** 2006-03-20

**Authors:** Irena Kostova, Georgi Momekov, Tzvetomira Tzanova, Margarita Karaivanova

**Affiliations:** ^1^Department of Chemistry, Faculty of Pharmacy, Medical University, 2 Dunav Street, Sofia 1000, Bulgaria; ^2^Department of Pharmacology and Toxicology, Faculty of Pharmacy, Medical University, 2 Dunav Street, Sofia 1000, Bulgaria

## Abstract

Complexes of lanthanum(III) with bis-coumarins: 3,3′-benzylidene-bis(4-hydroxy-2H-1-benzopyran-2-one) (H_2_L1) and bis(4-hydroxy-2-oxo-2H-chromen-3-yl)-(1H-pyrazol-3-yl)-methane (H_2_L2) were synthesized by reaction of lanthanum(III) salt and the ligands, in amounts equal to metal : ligand molar ratio of 1 : 2. The complexes were prepared by adding an aqueous solution of lanthanum(III) salt to an aqueous solution of the ligand subsequently raising the pH of the mixture gradually to circa 5.0 by adding dilute solution of sodium hydroxide. The lanthanum(III) complexes with bis-coumarins were characterized by different physicochemical methods—elemental analysis, IR-, ^1^H-, and ^13^C-NMR-spectroscopies, and mass spectral data. The spectral data of lanthanum(III) complexes were interpreted on the basis of comparison with the spectra of the free ligands. This analysis showed that in the La(III) complexes, the ligands coordinated to the metal ion through both deprotonated hydroxyl groups. On the basis of the *ν*(C=O) red shift observed, participation of the carbonyl groups in the coordination with the metal ion was also suggested. In the present study, we performed a cytotoxic-effects screening of the lanthanum complexes with H_2_L1 and H_2_L2 in a panel of human tumor cell lines, using the standard MTT-dye reduction assay for cell viability. The panel consisted of the acute myeloid leukemia-derived HL-60 and the chronic myeloid leukemia-derived BV-173. Following a 24- hour treatment of BV-173 cells with lanthanum complex of H_2_L1 at 100 or 200 *μ*M led to a DNA-laddering. The findings suggest that the observed cytotoxicity of the lanthanum complex of H_2_L1 on BV-173 is at least partly mediated through induction of programmed cell death.

## INTRODUCTION

The coumarins constitute an important class of compounds, with several types of pharmacological agents possessing anticancer, anti-HIV, anticoagulant, and spasmolytic and antibacterial activity among others. Of the many actions of coumarins, antioxidant and antiproliferative effects stand out. A large number of structurally novel coumarin derivatives has ultimately been reported to show substantial cytotoxic activity in vitro and in vivo.

Subsequent analysis of scientific literature revealed numerous reports on the antiproliferative and antitumor activities of a variety of coumarin compounds, for example, both coumarin itself and 7-hydroxycoumarin have been reported to inhibit the proliferation of a number of human malignant cell lines in vitro [1–4] and have demonstrated activity against several types of animal tumors [5–9]. These compounds have also been reported in clinical trials to demonstrate activity against prostate cancer, malignant melanoma, and metastatic renal cell carcinoma [10–12].

For coumarins, generally the in vitro structure-activity relationship studies have shown that cytotoxicity is found with derivatives containing ortho-dihydroxy substituents. Also, the chemical-structure/biological activity study of the coumarins showed that the addition of a cathecolic group to the basic structure induces increased cytotoxic activity in tumor cell lines [13]. The different cytotoxic values found for the coumarins could be related to the presence and the positions of the hydroxyls in their structures.

The complexes of rare earth ions have aroused much interest. Lanthanides are a subject of increasing interest in bioinorganic and coordination chemistry [14, 15].

Nowadays, a lot of studies report complexes of coumarin derivatives with rare earth metals, which possess biological activity. Thus, lanthanide complexes of 3-sulfo-4-hydroxycoumarin [16] and bis(4-hydroxy-3-coumarinyl)-acetic acid [17] have been synthesized and characterized. The complexes have revealed good anticoagulant action.

Lanthanides manifest an antitumor activity. Furthermore, literature data show that the coumarins have also these properties. These previous data from literature are in accordance with our investigations. They give us a reason to suppose that complexes of coumarins with lanthanum could present interesting metalorganic compounds with antitumor activity. As a result from our earlier work, the cytotoxic profile of some complexes of mendiaxon, warfarin, coumachlor, and niffcoumar with lanthanides against P3HR1, K-562, and THP-1 cell lines was proved [18–23]. With the relatively resistant CML-derived erythroleukemic K-562 cell line, we obtained very interesting in vitro results. It is noteworthy that the lanthanide complexes with niffcoumar have pronounced cytotoxic effects. They have strong cell proliferation inhibiting effects (only about 30% of the cells were survival). This means that the resistant tumor cells may be inhibited significantly with lanthanide complexes. This means also that the spectrum of cytotoxicity of these complexes is different from cis-DDP(II) and from Pt(II) complexes. These results are of some interest as a possibility to influence resistant tumors. The corresponding lanthanide salts are found to be of very low or missing activity. So far, we can conclude that the structure metal-ligand determines the antitumor spectrum of the new complexes. Those in vitro effects are not so clearly expressed as it is in the case of cis-DDP(II). Nevertheless their studying is interesting in connection with other cell lines and tumors in order to find out the differences in their spectrum of activity.

Little is known about the complexing ability of lanthanum(III) with coumarins. A survey of the literature reveals that no work has been done on the reactions of lanthanum(III) with H_2_L1 and its derivatives. It was, therefore, considered worthwhile to study the complexation, and in the first place, the objective of this study was to determine whether the new complexes were active as cytotoxic agents.

In the present study, we perform investigation of the coordination ability of H_2_L1 and H_2_L2 in complexation reaction with lanthanum(III). The obtained La(III) complexes with these coumarin ligands were characterized by elemental analysis, physicochemical methods, and mass-, NMR-, and IR-spectroscopy. The complicated vibrational spectra of lanthanum(III) complexes were interpreted on the basis of comparison with the vibrational spectra of the free ligands. The most sensitive to coordination modes of the ligands have been assigned and discussed.

We observed that La(III) possesses a cytotoxic activity, and literature data show that the coumarins have also these properties. That is why our synthesis of complexes of La(III) is taken into consideration with cytotoxic screening and further pharmacological study.

## EXPERIMENTAL

### Chemistry

**Scheme 1:**
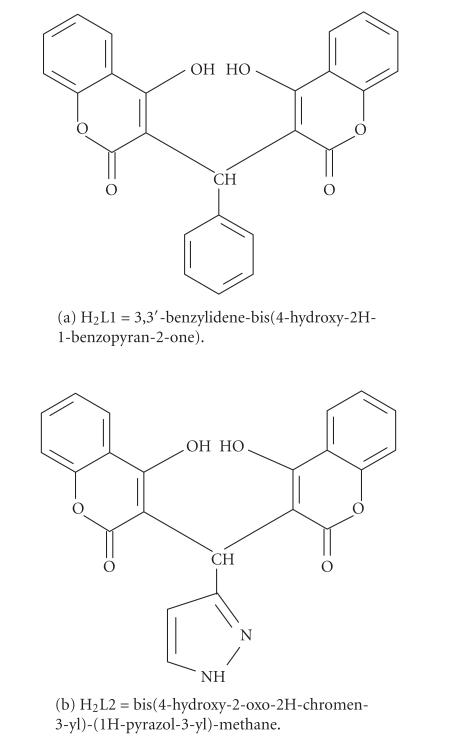
Structures of the ligands.

The compounds used for preparing the solutions were Merck products, p.a. grade: La(NO_3_)_3_ · 6H_2_O. H_2_L1 and H_2_L2 were used for the preparation of metal complexes as ligands (Scheme 1). The synthesis of the ligands was described recently by us [24].

The complexes of lanthanum(III) with H_2_L1 and H_2_L2 were synthesized by reaction of lanthanum(III) salt and the ligand, in amounts equal to metal : ligand molar ratio of 1 : 2. The ligands were insoluble in water. On raising the pH by the dropwise addition of a dilute solution of sodium hydroxide (0.1 mol L^−1^), the ligands were dissolved. The complexes were prepared by adding aqueous solution of Ln(III) salts (1 mmol) to the solution of the respective ligand (2 mmol). The reaction mixtures were stirred with an electromagnetic stirrer at 25°C for one hour. At the moment of mixing of the solutions, precipitates were obtained. The precipitates were filtered, washed several times with water, and dried in a desicator to constant weight. Yields are: La(L1) (OH) · H_2_O (94%); La(L2) (OH) · 2H_2_O (89%).

The complexes were insoluble in water, slightly soluble in methanol and ethanol, and well soluble in DMSO.

The carbon, hydrogen, and nitrogen contents of the compounds were determined by elemental analysis.

The water content was determined by Metrohn Herizall E55 Karl Fisher Titrator.

IR spectra (Nujol) were recorded on spectrophotometers FTIR-8101M Shimadzu (3800–400 cm^−1^) and Perkin-Elmer GX auto image system (700–200 cm^−1^).

^1^H-NMR spectra were recorded at room temperature on Brucker WP 250 (250 MHz) spectrometer in DMSO-d_6_. Chemical shifts are given in ppm.

^13^C-NMR spectra were recorded at ambient temperature on Brucker 250 WM (62.9 MHz) spectrometer in DMSO-d_6_. Chemical shifts are given in ppm, downfield from TMS.

Mass spectra were recorded on a Jeol JMS D 300 double focusing mass spectrometer coupled to a JMA 2000 data system. The compounds were introduced by direct inlet probe, heated from 50°C to 400°C at a rate of 100°C/min. The ionization current was 300 mA, the accelerating voltage 3 kV, and the chamber temperature 150°C.

### Pharmacology

In the present study, we performed a cytotoxic-effects screening of the lanthanum complexes with H_2_L1 and H_2_L2 in a panel of human tumor cell lines, using the standard MTT-dye reduction assay for cell viability. The panel consisted of the acute myeloid leukemia-derived HL-60 and the chronic myeloid leukemia-derived BV-173.

In order to shed some light over the mechanistic particulars engaged in the cytotoxic mode of action of the new lanthanum complexes, we carried out DNA-isolation and gel electrophoresis to evaluate the ability of the more active lanthanum complex with H_2_L1 to trigger programmed cell death.

*Cell culture maintenance, drug solutions, and treatment*

The human tumor cell lines used in the present study were supplied from the Department of Human and Animal Cell Cultures at the German Collection of Microorganisms and Cell Cultures. They were maintained as suspension—type cultures at standard conditions: humidified atmosphere with 5% carbon dioxide, at 37°C in a “Heraeus” incubator, using RPMI-1640 medium, supplemented with 10% heat-inactivated fetal calf serum and 2 mM L-glutamine. Cells were kept in log phase via replacement of cellular suspension aliquots by fresh medium two or three times weekly.

Stock solutions of the new investigated lanthanum complexes (200 mM) were freshly prepared in DMSO and diluted with RPMI-1640 in order to obtain the desired final concentrations. Less than 1% of the solvent was available at the final dilutions obtained.

All of the procedures concerning the cell culture maintenance, drug dissolution, and treatment were carried out in a “Heraeus” Laminar flow cabinet.

*Cell viability determination (MTT assay)*

The MTT-dye reduction assay was carried out as described by Mosmann [25] with some modifications [26]. Briefly, exponentially growing cells were seeded in 96-well plates (100 *μ*L aliquots/well) at a density of 1 × 10^5^ cells/mL. After a 24 hours incubation at 37°C, they were exposed to either the lanthanum complex of H_2_L1 or the lanthanum complex with H_2_L2 (12.5–200 *μ*M) for 72 hours. After the incubation period expired, 10 *μ*L aliquots of MTT solution (10 mg/mL in PBS) were added to every well. Thereafter the plates were incubated for 4 hours at 37°C and the formazan crystals formed were dissolved through addition of 100 *μ*L/well 5% formic acid in 2-propanol (Merck). The absorption of the samples was measured using an ELISA reader (Uniscan Titertec) at wavelength of 580 nm. The blank consisted of 100 *μ*L RPMI 1640 medium (Sigma), 10 *μ*L MTT stock, and 100 *μ*L 5% formic acid in 2-propanol.

*DNA isolation and gel electrophoresis*

The DNA extraction and horizontal gel electrophoresis procedures were executed as previously described [26]. About 5 × 10^6^ treated and untreated BV-173 cells were washed in PBS. Cell pellets were re-dispersed in 0.25 mL PBS and lysed through addition of 0.5 mL buffer containing 0.5% Triton X-100, 20 mM Tris-HCL, and 1 mM EDTA (pH = 7.4). Samples were incubated on ice for 5 minutes and then centrifuged at 13 000 rpm for 20 minutes. The supernatants were transferred into 2 mL Eppendorf safe lock tubes and then 0.937 mL 2-propanol as well as 0.187 mL 6M solution of NaCl were added to each sample. The tubes were gently agitated and incubated at −20°C overnight in order to allow DNA precipitation. The samples were centrifuged for 20 minutes at 13 000 rpm, the supernatants were decanted and DNA was washed in 1 mL ice cold 70% ethanol and then air dried. The isolated DNA was dissolved in 20 *μ*L purified water and analyzed by gel electrophoresis in 0.8% agarose gel. Finally DNA was visualized by means of ethidium bromide staining and UV transillumination and photographed with a fixed digital camera (Bio Doc IT™ system).

*Statistics*

The data processing included the Student *t* test with *p* ≤ 0.05 taken as significance level, using Microsoft EXCEL for PC.

## RESULTS AND DISCUSSION

### Chemistry

The complexes were characterized by elemental analysis. The metal ion was determined after mineralization. The water content in the complexes was determined by Karl Fisher analysis. The formation of the complexes was confirmed by IR-spectroscopy, ^1^H, ^13^C-NMR-spectrometry, and mass spectral data.

Table 1 shows the data of the elemental analysis of the complexes serving as a basis for the determination of their empirical formulae. The elemental analysis data of the La(III) complexes obtained are in agreement with the presented formulas.

The suggested formulas were further confirmed by mass spectral fragmentation analysis. As it is seen from Table 2, the first peaks in the La(III) complexes spectra (although with low intensity) correspond to the mass weight of the complex formation (La(L1) (OH) · H_2_O and La(L2) (OH) · 2H_2_O, resp) and the next ones to that of the ligands. The results thus obtained are in agreement with metal : ligand ratio 1 : 1 in the investigated complexes. The data of mass spectral fragmentation of the ligands and of the complexes are presented in Table 2.

*IR spectra of the complexes*

The mode of bonding of the ligands to La(III) was elucidated by recording the IR spectra of the complexes as compared with that of the free ligands.

IR spectra of the compounds were recorded on solid state in Nujol in the range 3800–400 cm^−1^. The data of the IR spectra of H_2_L1, H_2_L2, and of the lanthanum complexes with these ligands are presented in Table 3.

*IR Spectrum of the lanthanum complex of* H_2_L1

The bands appear in the IR spectrum of H_2_L1 at 3074, 3032; 1660, 1617; 1605, 1568; 1496, 1182, 1160, 1092, 1074 cm^−1^. The bands at 1660 and 1617 cm^−1^ can be attributed to the stretching vibrations of the carbonyl groups of the lacton rings. Bands at 1605 and 1568 cm^−1^ can be related to the stretching vibrations of the conjugated olefinic system. The vibrations at 1496 cm^−1^ correspond to the aromatic systems.

A broad band, characteristic of *ν*_OH_ of coordinated water, was observed in the range 3300–3400 cm^−1^ in the spectrum of the complex. The weak bands observed at 3074 and 3032 cm^−1^ in the spectrum of the free ligand are missing in the spectrum of the complex. A comparison of the infrared spectra of the ligand and of the complex reveals the disappearance of absorption bands observed in the free ligand at 3074, 3032 cm^−1^ and 1345, 1336 cm^−1^ associated with the stretching and deformation OH of the phenolic groups, indicating the loss of phenolic protons on complexation, thus forming metal-oxygen bonds, which appear as bands in the far IR region.

The *ν*_C=O_ bands at 1660 and 1617 cm^−1^ exhibit a shift of 30–40 cm^−1^ to lower wavenumber values on complexation, which may be taken as evidence for the participation of the C=O groups in coordination.

The C−C and C−O stretch and the C−O−C band are all shifted in the complex. Similar frequency shifts are observed for the other complexes and are attributed to complexation of the positive ion with the carbonyl oxygen [27].

*IR Spectrum of the lanthanum complex of* H_2_L2

The bands appear in the IR spectrum of H_2_L2 at 3139, 3070; 1669, 1635; 1610, 1539; 1496, 1187, 1150, 1110, 1044 cm^−1^. The bands at 1669 and 1635 cm^−1^ can be attributed to the stretching vibrations of the carbonyl groups of the lacton rings. Bands at 1610 and 1539 cm^−1^ can be related to the stretching vibrations of the conjugated olefinic system. The vibrations at 1496 cm^−1^ correspond to the aromatic systems. Bands at 1620–1417 cm^−1^ can be attributed to the stretching vibrations of pyrazol and they remain almost the same in the complex.

A broad band, characteristic of *ν*_OH_ of coordinated water, was observed in the range 3300–3400 cm^−1^ in the spectrum of the complex. The weak bands observed at 3139 and 3070 cm^−1^ in the spectrum of the free ligand are missing in the spectrum of the complex. A comparison of the infrared spectra of the ligand and of the complex reveals the disappearance of absorption bands observed in the free ligand at 3139, 3070 cm^−1^ and 1360, 1300 cm^−1^ associated with the stretching and deformation OH of the phenolic groups, indicating the loss of phenolic protons on complexation, thus forming a metal-oxygen bonds, which appear as bands in the far IR region.

The *ν*_C=O_ bands at 1669 and 1635 cm^−1^ exhibit a shift of 20–30 cm^−1^ to lower wavenumber values on complexation, which may be taken as evidence for the participation of the C=O groups in coordination.

The C−C and C−O stretch and the C−O−C band are all shifted in the complex. Similar frequency shifts are observed for the other complexes and are attributed to complexation of the positive ion with the carbonyl oxygen [27].

IR spectra of the compounds were recorded on solid state in Nujol in the range 700–220 cm^−1^. The spectra of the complexes showed new bands, in comparison with these of the free ligands, which have been assigned to the rocking, waggling, and metal-oxygen stretching vibrations.

^1^H- *and* ^13^C-NMR *spectra of the ligands and their* La(III) *complexes*

Metal ion coordination with ligand by means of oxygen atoms of C=O groups and of the deprotonated hydroxyl groups was shown owing to data of ^1^H and ^13^C-NMR spectra.

Proton spectra of the compounds recorded at 250 MHz in DMSO-d_6_ confirmed the formation of the complex. The typical chemical shifts of the ^1^H-NMR spectra in DMSO-d_6_ are presented in Table 4. As it is seen from Table 4, different chemical shifts were observed in the complexes and these changes were attributed to coordination of the ligands to La(III).

^13^C-NMR spectra of the ligands and of the complexes were recorded at 62.9 MHz in DMSO-d_6_. The results of ^13^C-NMR spectra of the compounds in *δ*d (ppm) are presented in Table 5.

The ligand H_2_L1 showed seven signals in the ^13^C-NMR spectra resonating at *δ* 131.91, 128.08, 126.70, 125.58, 123.92, 123.76, and 115.95 ppm for thirteen methine carbons (Table 5). In agreement with literature data, the peaks at *δ* 131.91, 123.92, 123.76, and 115.95 ppm were related to C-7, C-5, C-6, and C-8 (the atom numbering is in agreement with the scheme in Table 4) carbons, respectively, of the coumarin moieties. The signals at *δ* 128.08, 126.70, and 125.58 ppm were assigned to C-3′ (and C-5′), C-4′, and C-2′ (and C-6′) carbons of the phenyl ring. The chemical shifts at *δ* 165.36, 164.87, 152.23, 139.94, 117.96, and 104.13 ppm are due to the C-2, C-4, C-8a, C-1′, C-4a, and C-3 quaternary carbons, respectively. Due to electron transfer from the hydroxyl and carbonyl oxygen atoms to La(III), a difference in chemical shifts was observed for the neighboring C-4, C-3, and C-2 carbon atoms of the complex and they confirmed the expected coordination of the ligand through both deprotonated hydroxyl and carbonyl oxygen atoms. The other carbon atoms were only slightly affected from the coordination of the metal. Similar chemical shifts were observed for the other ligand and its complex (Table 5). On the basis of the results thus obtained, it was suggested that the ligands act as tetradentate ones in the La(III) complex formation.

**Scheme 2:**
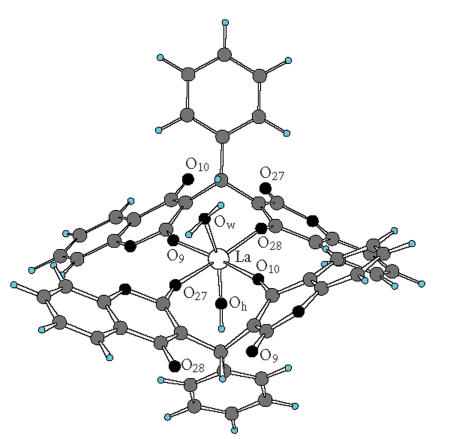
Suggested metal-ligand coordination in the investigated La(III) complexes.

In our previous investigation, we have performed accurate density functional theory study (DFT) of the neutral and deprotonated ligands [28]. The results obtained showed that the most probable reactive sites for electrofilic attack (metal binding) in the L^2−^ are the carbonylic and the deprotonated hydroxylic oxygen atoms. Our IR and NMR spectral data confirmed that the carbonylic and deprotonated hydroxylic oxygen atoms are included in the coordination to the metal ion in the complexes studied, as described in the previous sections. Further, molecular modeling was performed to obtain the most probable molecular geometry of the complex. Our calculations have showed that it is unfavorable for one metal ion to coordinate to all four oxygen atoms of one ligand. Thus, on the basis of the experimental and theoretical results, we were able to suggest that in the metal complex, the metal ion coordinates to the carbonylic (O_9_) and deprotonated hydroxylic oxygen atoms (O_28_) from one ligand and through the carbonylic (O_27_) and deprotonated hydroxylic oxygen atoms (O_10_) from a second ligand, as it is shown in Scheme 2. The other carbonylic (O_27_) and deprotonated hydroxylic oxygen atoms (O_10_) from the first ligand will coordinate with another metal ion, as well, the carbonylic (O_9_) and deprotonated hydroxylic oxygen atoms (O_28_) from the second ligand will coordinate to the next metal ion, forming a polymeric structure. The other two coordination sites of the metal ion (Scheme 2) are occupied from an OH group and a water molecule. Thus, the metal ion is 6 coordinate, the ligand is tetradentate, and the metal : ligand ratio is 1 : 1, as the experimental results showed.

### Pharmacology

*In vitro cytotoxicity*

The spectrophotometric data regarding the MTT-dye reduction assay are summarized in Table 6.

**Figure 1:**
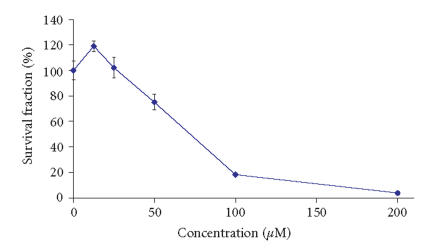
Cytotoxic effects of La-1 on HL-60 cells as assessed by the MTT-dye reduction assay following a 72- hour treatment. Each data point represents the arithmetic mean ± standard deviation (error bars) of at least 6 independent experiments.

**Figure 2:**
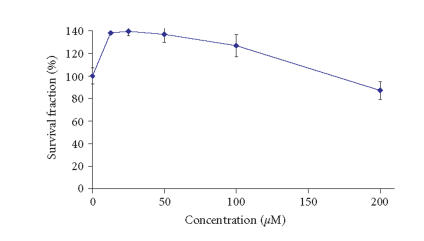
Cytotoxic effects of La-2 on HL-60 cells as assessed by the MTT-dye reduction assay following a 72- hour treatment. Each data point represents the arithmetic mean ± standard deviation (error bars) of at least 6 independent experiments.

**Figure 3:**
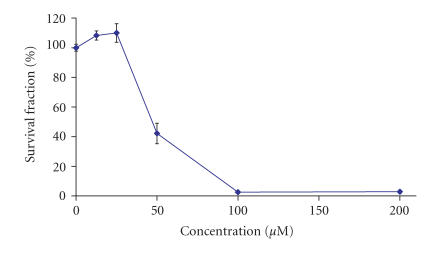
Cytotoxic effects of La-1 on BV-173 cells as assessed by the MTT-dye reduction assay following a 72- hour treatment. Each data point represents the arithmetic mean ± standard deviation (error bars) of at least 6 independent experiments.

**Figure 4:**
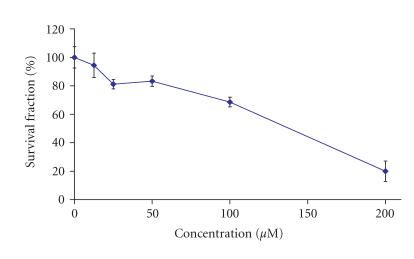
Cytotoxic effects of La-2 on BV-173 cells as assessed by the MTT-dye reduction assay following a 72- hour treatment. Each data point represents the arithmetic mean ± standard deviation (error bars) of at least 6 independent experiments.

The newly-synthesized lanthanum complexes exhibited cytotoxic effects against HL-60 and BV-173 human leukemic cell, that enabled the construction of concentration-response curves, as presented in Figures 1–4. In order to allow comparison of their relative potencies, the IC50 values were extrapolated from the concentration-response curves; these are summarized on Table 7.

As evident from the concentration-response curve on Figure 1, the lanthanum complex of H_2_L1 failed to induce any significant effects at concentrations less than 25 *μ*M and even certain stimulation of malignant cell growth was encountered at the lowest concentration. At a concentration of 50 *μ*M, however, the investigated compound reduced the viable cells by circa 25%, whereas at 100 *μ*M, the vital cells were less than 20%. At the highest concentration exploited in our experimental system (200 *μ*M), an almost total eradication of the malignant cells was encountered with less than 4% survival fraction. The other complex exerted marginal cytotoxic effect on HL-60 with certain stimulation of cellular proliferation at concentrations up to 100 *μ*M and small reduction of the cell viability by circa 13% at the highest concentration of 200 *μ*M.

The data obtained from the evaluation of the cytotoxic activity of the lanthanum complex of H_2_L1 on BV-173 cells is shown in Figure 3. As evident from the concentration-response curve, it caused a small increase of cell viability at the lowest concentrations of 12.5 and 25 *μ*M by circa 8 and 10%, respectively. At the higher concentrations of 50 *μ*M, the cell survival fraction was reduced by circa 58%, whereas at concentrations higher than 100 *μ*M, the vital cells were less than 3%. The lanthanum complex with H_2_L2 also caused concentration-dependent cytotoxic effect on BV-173 cells, although less pronounced than that of the lanthanum complex of H_2_L1 in respect to both relative potency and maximal efficacy encountered. At concentrations of 25 and 50 *μ*M, the lanthanum complex with H_2_L2 reduced the viable cells by circa 20%, whereas at the higher concentration of 100 *μ*M, it caused circa 31% inhibition of malignant cell proliferation. The treatment of BV-173 cells with 200 *μ*M of the lanthanum complex with H_2_L2 resulted in circa 80% reduction of cell viability.

In contrast to the observed effects of the lanthanum complexes, the corresponding lanthanum(III) nitrate was practically devoid of cytotoxic effects at the same experimental conditions [18–24].

*DNA-fragmentation analysis*

**Figure 5:**
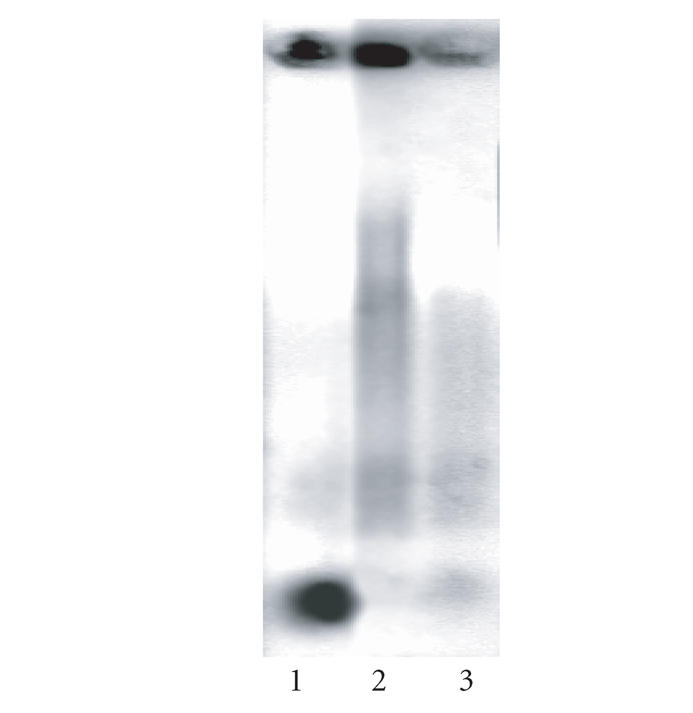
Agarose gel electrophoresis of DNA, isolated from the cytosolic fraction of BV-173 cells as follows: untreated control (lane 1), following a 24- hour treatment with La-1 at 200 *μ*M (lane 2) or 100 *μ*M (lane 3).

Following a 24 hours treatment of BV-173 cells with lanthanum complex of H_2_L1 at 100 or 200 *μ*M led to an DNA-laddering, that is indicative for the programmed cell death (Figure 5). These findings suggest that the observed cytotoxicity of the lanthanum complex of H_2_L1 on BV-173 is at least partly mediated through induction of programmed cell death.

## CONCLUSIONS

The coordination ability of the ligands has been proved in complexation reaction with lanthanum(III) ion. The elemental analysis and mass spectral data confirmed the compositions of the compounds. ^1^H-, ^13^C-NMR-, and IR-spectral analysis of the ligands and their La(III) complexes confirmed the suggested coordination of the ligands through both the hydroxyl and carbonyl oxygen atoms.

In our hands, the two novel lanthanum complexes under investigation exhibited in vitro cytotoxic effects in micromolar concentrations. The lanthanum complex with H_2_L1, however, demonstrated far more pronounced cytotoxic effects as compared to the lanthanum complex with H_2_L2. On the basis of the observed considerable cytotoxic activity of lanthanum complex with H_2_L1 on HL-60 and BV-173 human leukemic cells, together with its documented ability to trigger programmed cell death, it could be concluded that lanthanum complex with H_2_L1 deserves further detailed pharmacological and toxicological evaluation.

According to our expectations, the complexes of lanthanum(III) possess a cytotoxic activity and their in vitro effects are clearly expressed. These results confirmed our previous observations on the cytotoxicity of lanthanum(III) complexes.

## Figures and Tables

**Table 1:** Elemental analysis data for La(III) complexes with bis-coumarins.

	Found/calculated				

Complex	%C	%H	%N	%H_2_O	%La

La(L1)(OH) · H_2_O	51.81	3.32	—	2.85	24.23
	51.37	2.91	—	3.08	23.80
La(L2)(OH) · 2H_2_O	44.94	3.05	5.07	6.35	23.22
	44.59	2.87	4.73	6.08	23.48

L_1_ = C_25_H_14_O_6_^2−^

L_2_ = C_22_H_12_N_2_O_6_^2−^.

**Table 2:** Mass spectral data of bis-coumarins and their La(III) complexes.

Ligand	M/z	(%)	Complex	M/z	(%)

H_2_L1=C_25_H_16_O_6_	412	8	La(L_1_)(OH) · H_2_O	586	8
	249	100		410	35
	221	17		305	100
	162	20		176	100
	120	37		—	—
H_2_L2=C_22_H_14_N_2_O_6_	402	0	La(L_2_)(OH) · 2H_2_O	596	1
	241	16		490	5
	240	100		460	3
	162	72		410	1
	120	74		307	65
	92	98		176	100

**Table 3:** Selected experimental IR frequencies of the ligands and their La(III) complexes (cm^−1^).

Compound	*ν* OH/H_2_O	*ν*(C=O)	*ν*(C=C)	*ν*(Py)	*ν*(Ar)	*δ*(COH)	*ν*(C−O)	

	—	—	—	—	—	—	1182 m	—
	3074 m	1660 s	1605 s	—	1496 m	1345 m	1160 m	772
H_2_L1=C_25_H_16_O_6_	3032 m	1617 s	1568 s	—	—	1336 m	1092 s	750
	—	—	—	—	—	—	1074 m	—
	—	—	—	—	—	—	1192 w	—
	3391 br	1620 sh	1508 s	—	1446 m	—	1150 w	757
La(L_1_)(OH) · H_2_O	—	1600 s	—	—	—	—	1109 m	—
	—	—	—	—	—	—	1109 m	—
	—	—	—	—	—	—	1094 w	—
	—	—	—	1620	—	—	1187 m	—
	3139 m	1669 s	1610 s	1559	1496 m	1360 m	1150 m	770
H_2_L2=C_22_H_14_N_2_O_6_	3070 m	1635 s	1539 s	1507	—	1300m	1110 s	748
	—	—	—	1417	—	—	1044 m	—
	—	—	—	1622	—	—	1194 w	—
	3379 br	1653 sh	1520 s	1560	1460 m	—	1145 w	759
La(L_2_)(OH) · 2H_2_O	—	1599 s	—	1508	—	—	1109 m	—
	—	—	—	1418	—	—	1054 w	—

^a^ br-broad, s-strong, m-medium, sh-shoulder, w-weak.

**Table 4:**
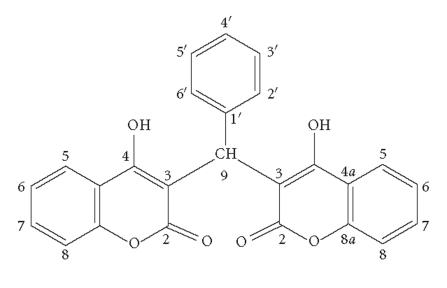
^1^H-NMR spectral shifts, *δ* (ppm) of the ligands and their La(III) complexes (250 MHz, DMSO-d_6_).

Compound	*δ* (ppm)		
	H_5_–H_8_^a^	H_9_^a^	H_2′_–H_6′_^a^

H_2_L1=C_25_H_16_O_6_	7.11–7.39	6.37	7.56–7.92
La(L_1_) (OH) · H_2_O	6.98–7.26	6.27	7.37–7.83
H_2_L2=C_22_H_14_N_2_O_6_	7.23–7.55	6.36	7.83–8.15
La(L_2_) (OH) · 2H_2_O	7.03–7.51	5.79	7.81–8.00

**Table 5:** ^13^C-NMR spectral shifts, *δ* (ppm) of the ligands and their La(III) complexes (62.9 MHz, DMSO-d_6_).

Atom	*δ* (ppm)			
	H_2_L1	La(L_1_) (OH) · H_2_O	H_2_L2	La(L_2_) (OH) · 2H_2_O

C-2	165.3	164.6	167.9	170.0
C-4	164.9	152.7	163.9	154.8
C-8a	152.2	152.5	152.7	154.6
C-1′	139.9	142.4	150.5	152.6
C-7	131.9	130.9	134.3	131.1
C-3′	128.1	127.7	131.6	130.0
C-5′	128.1	127.7	—	—
C-4′	126.7	126.6	—	—
C-6′	125.6	124.8	—	—
C-2′	125.6	124.8	126.1	128.0
C-5	123.9	124.1	124.3	122.0
C-6	123.8	122.9	123.3	121.5
C-4a	117.9	119.9	119.5	120.2
C-8	115.9	115.4	115.8	115.1
C-3	104.1	103.4	101.4	103.0
C-9	35.9	37.5	30.1	33.1

**Table 6:** Spectrophotometric data from MTT assay, concerning the cytotoxic effects of the newly synthesized lanthanum complexes with 3,3′-benzylidene-bis(4-hydroxy-2H-1-benzopyran-2-one) (La-1) and bis(4-hydroxy-2-oxo-2H-chromen-3-yl)-(1H-pyrazol-3-yl)-methane (La-2).

Cell line complex	MTT-formazan absorption at 580 nm						
		Untreated control	12.5 *μ*M	25 *μ*M	50 *μ*M	100 *μ*M	200 *μ*M

HL-60	La-1	1.22 ± 0.089	1.203±0.058	0.707±0.100	0.705±0.092	0.446±0.090	0.114±0.031
	La-2	0.737±0.052	1.017±0.007	1.028±0.007	1.008±0.051	0.935±0.071	0.643±0.058
BV-173	La-1	0.957±0.023	1.036±0.029	1.053±0.059	0.403±0.066	0.024±0.008	0.027±0.009
	La-2	1.137±0.096	1.464±0.059	1.127±0.063	1.061±0.042	0.909±0.058	0.859±0.095

**Table 7:** IC_50_ values of the lanthanum complexes with 3,3′-benzylidene-bis(4-hydroxy-2H-1-benzopyran-2-one) (La-1) and bis(4-hydroxy-2-oxo-2H-chromen-3-yl)-(1H-pyrazol-3-yl)-meth- ane (La-2), derived from the corresponding concentration-response curves.

Cell line	IC_50_ value (*μ*M)	
	La-1	La-2

HL-60	71.92	> 200
BV-173	46.89	138.03
